# Metformin's impact on asprosin and FBN1 expression: Potential mechanisms beyond insulin sensitivity in type 2 diabetes in rats

**DOI:** 10.1016/j.crphar.2024.100207

**Published:** 2024-12-09

**Authors:** Ali Dashtkar, Mansour Karajibani, Mohsen Saravani, Roya zanganeh, Hamed Fanaei

**Affiliations:** a Department of Nutrition, School of Medicine, Zahedan University of Medical Sciences, Zahedan, Iran; b Health Promotion Research Center, Department of Nutrition, School of Medicine, Zahedan University of Medical Sciences, Zahedan, Iran; c Genetics of Non-communicable Disease Research Center, Zahedan University of Medical Sciences, Zahedan, Iran; d Department of Clinical Biochemistry, School of Medicine, Zahedan University of Medical Sciences, Zahedan, Iran; e Pregnancy Health Research Center, Zahedan University of Medical Sciences, Zahedan, Iran; f Department of Physiology, School of Medicine, Zahedan University of Medical Sciences, Zahedan, Iran

**Keywords:** Metformin, Diabetes mellitus, Rats, Asprosin, FBN1

## Abstract

**Background:**

Asprosin, a novel adipokine released under fasting conditions, may play a significant role in the pathophysiology of type 2 diabetes mellitus (T2DM). The objective of this study is to investigate the effects of metformin on serum asprosin levels and FBN1 gene expression in white adipose tissue in male rats.

**Methods:**

Thirty-two male Wistar rats were randomly and equally divided into four groups (n = 8): 1. Control Group (CON): Received standard food; 2. Non-Diabetic Metformin Group (CON + MET): Received standard food and were treated with metformin (400 mg/kg/day) for four weeks; 3. Diabetic Group (DM): Induced with T2DM; and 4. Diabetic Metformin Group (DM + MET): Induced with T2DM and treated with metformin (400 mg/kg/day) for four weeks. Finally, serum asprosin levels, lipid profiles, fasting glucose, and insulin concentrations were measured. The expression level of the FBN1 gene in white adipose tissue was quantified using quantitative real-time polymerase chain reaction (qRT-PCR).

**Results:**

Serum asprosin levels were significantly higher in the DM group compared to both the CON and CON + MET groups (P < 0.0001). However, serum asprosin levels were significantly lower in the DM + MET group than in the DM group (P = 0.0003). Additionally, the FBN1 gene expression level in white adipose tissue was significantly higher in the DM group compared to the CON group (P = 0.0053), while FBN1 gene expression was significantly lower in the DM + MET group than in the DM group (P < 0.0001). Furthermore, lipid profile, insulin resistance, and fasting blood sugar improved in the CON + MET and DM + MET groups compared to the CON and DM groups, respectively.

**Discussion:**

Our findings in diabetic male rats reveal that metformin treatment significantly downregulates FBN1 gene expression and reduces serum asprosin levels, suggesting a potential mechanism for its therapeutic benefits beyond improving insulin sensitivity.

## Highlights

• The objective of this study is to examine the impact of metformin on serum levels of asprosin and FBN1 gene expression in white fat tissue in male rats.
• FBN1 gene expression and serum asprosin level in the diabetic group treated with metformin was significantly lower than the diabetic group.
• These finding suggest therapeutic benefits beyond insulin sensitivity for Metformin.

## Graphical abstract

**Figure:**
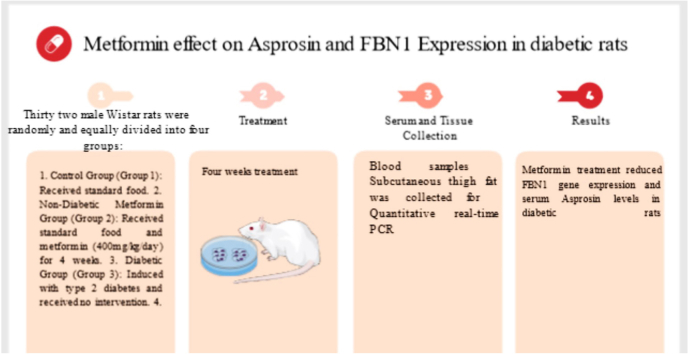

## Abbreviations

qRT-PCR: quantitative Real-time polymerase chain reaction
OLFR734: Olfactory receptor 734
CREB: CAMP response element-binding protein
CAMP: Cyclic adenosine monophosphate
PKA: Protein kinase A
TLR4: Toll-like receptor 4
JNK: c-Jun N-terminal kinase
STZ: Streptozotocin
HDL: High-density lipoprotein
LDL: Low-density lipoprotein

## 1 Introduction

Diabetes is a chronic metabolic disorder characterized by high blood glucose levels resulting from absolute or relative insulin deficiency, in the context of beta-cell dysfunction, insulin resistance, or both (Cole and Florez, 2020). This disease is currently classified into type 1 diabetes mellitus, type 2 diabetes mellitus (T2DM), "other," and gestational diabetes based on cause and pathology (CDC, 2024). Over 90% of diabetes mellitus cases are T2DM (Zheng et al., 2018). Genetic, environmental, and metabolic risk factors are interrelated and contribute to the development of T2DM. A strong family history of diabetes mellitus, advanced age, obesity, and physical inactivity place individuals at the highest risk (Fletcher et al., 2002). It is estimated that more than 600 million people worldwide are currently affected by obesity, which is associated with over 45 comorbidities, including several atherogenic disorders that make up metabolic syndrome (Lois and Kumar, 2009). The earlier onset of T2DM is associated with higher BMI, and the rising prevalence of overweight and obesity is the most important factor in the increasing number of younger people diagnosed with T2DM (Wild and Byrne, 2006). Therefore, identifying and investigating chemical biomarkers has a significant impact on diagnosing and treating diseases and risk factors related to diabetes.

One of the recently discovered biomarkers is asprosin (Zhang et al., 2019). Asprosin is a new adipokine discovered in 2016 by Romere et al. (2016). This adipokine, which is secreted during fasting, is coded by the FBN1 gene and results from the C-terminal cleavage of pro-fibrillin. Asprosin, secreted from white adipose tissue, affects the liver, activates the G protein–cyclic AMP (cAMP)–protein kinase A (PKA) pathway, and releases glucose into the blood (Romere et al., 2016; Nakhaei et al., 2019; Behrasi et al., 2022). As a result of FBN1 gene expression, pro-fibrillin is produced, which is processed by Furin protease to create 140-amino-acid asprosin and fibrillin-1 (Elnagar et al., 2022). In the hypothalamus, asprosin activates AgRP neurons and increases appetite through the G-cAMP-PKA protein pathway. In addition, the asprosin receptor in the liver, OLFR734, binds asprosin through the CREB pathway, leading to glucose production and release. Asprosin also has various roles; for example, in pancreatic β cells, it binds to TLR4, increasing inflammation and apoptosis of these cells, while also reducing insulin secretion by stimulating the TLR4/JNK-mediated pathway and inhibiting cAMP levels (Yuan et al., 2020). Metformin is a low-risk drug belonging to the biguanide group and is commonly used as the first-line treatment for T2DM (McCreight et al., 2016). Intestinal absorption of metformin may be mediated primarily by the plasma membrane monoamine transporter, expressed on the luminal side of enterocytes, and the drug is excreted unchanged in urine. Metformin lowers both basal and postprandial blood sugar. It also has other effects, such as improving insulin signaling, reducing fatty acid and triglyceride synthesis, and increasing beta-oxidation (Gong et al., 2012). Metformin may also increase glucose utilization in peripheral tissues and potentially decrease food intake. The mechanisms by which metformin works are complex. One key mechanism involves its effect on mitochondria; due to its positive charge, metformin accumulates at concentrations up to 1000 times higher inside the mitochondria than in the extracellular environment. By affecting complex I of the respiratory chain, metformin inhibits ATP production (Rena et al., 2017).

In this study, our hypothesis was that metformin influences serum asprosin levels by modulating the expression of the asprosin (FBN1) gene. Consequently, we evaluated not only serum asprosin levels but also FBN1 gene expression and other relevant biochemical parameters.

## 2 Materials and methods

### 2.1 Animal Model

Thirty-two adult male Wistar rats, with an average weight of 200–220 g, were used in this experimental study. The rats were purchased from the Laboratory Animal Research Center of Zahedan University of Medical Sciences. All animal procedures were conducted in accordance with the guidelines of the Ethics Committee for Working with Laboratory Animals of Zahedan University of Medical Sciences (Ethics Code: IR.ZAUMS.AEC.1401.014).

The rats were housed in the Laboratory Animal Research Center of Zahedan University of Medical Sciences under controlled environmental conditions: a 12-h light/12-h dark cycle, a temperature of 22–25 °C, and a humidity of 40–50%. The rats had ad libitum access to food and water. To allow the animals to acclimate to their new environment, the study commenced 7 days after they were housed in the laboratory.

### 2.2 Experimental design

Animal subjects were randomized into four groups(n = 8).

1 Control Group (CON): Received standard food for four weeks.
2 Non-Diabetic Metformin Group (CON + MET): Received standard food and metformin (400 mg/kg/day) for four weeks (Ismail et al., 2015).
3 Diabetic Group (DM): Induced with T2DM and received no intervention.
4 Diabetic Metformin Group (DM + MET): Induced with T2DM and received metformin (400 mg/kg/day) for four weeks.

### 2.3 High-fat diet Preparation

To provide a high-fat diet (HFD), 30% w/w tail fat from sheep, 3% w/w cholesterol, and 10% w/w sucrose were added to the usual pelleted diet for rats. This mixture was combined with ten times its weight in water and formed into a paste, then shaped into rat pellet form and air-dried (Rena et al., 2017). The rats were weighed weekly to ensure their growth.

### 2.4 Induction of type 2 diabetes

At the beginning of the study, for four weeks, rats in the CON and CON + MET groups were fed standard food. In addition, the DM and DM + MET groups were fed an HFD for four weeks. After this period, the DM and DM + MET groups received a low dose of streptozotocin (STZ) (45 mg/kg) to induce T2DM. Then, the DM and DM + MET groups were fed standard food. In addition, the CON + MET and DM + MET groups were treated with metformin for four weeks.

Rats with fasting blood glucose levels above 200 mg/dL were considered diabetic (Ismail et al., 2015).

### 2.5 Metformin administration

After being fed an HFD, the rats were gavaged with metformin at a dose of 400 mg/kg/day (Actoverco, Iran) for four weeks. For this purpose, metformin was mixed with a normal saline solution.

### 2.6 Blood sugar Measurement

Fasting blood sugar levels were measured from tail blood using a glucometer prior to tissue sampling.

### 2.7 Serum and tissue Collection

Blood samples were collected from the hearts of all rats to isolate serum. Subcutaneous thigh fat was collected for RNA extraction.

### 2.8 Serum biochemical analysis

Serum concentrations of asprosin (Zell Bio, Germany), insulin (Zell Bio, Germany), and lipid profiles (Pars Azmon, Iran) were analyzed using rat ELISA kits according to the manufacturer's instructions.

### 2.9 RNA Isolation

First, we homogenized the adipose tissue and then extracted the RNA using RNX-plus (Sinacolon, Iran) solution.

### 2.10 Complementary DNA (cDNA) synthesis

Following RNA extraction, we utilized the complementary DNA (cDNA) synthesis kit to transform the isolated RNAs into DNA.

### 2.11 Quantitative real-time polymerase chain reaction **(qRT-PCR)**

**Fig. 1:**
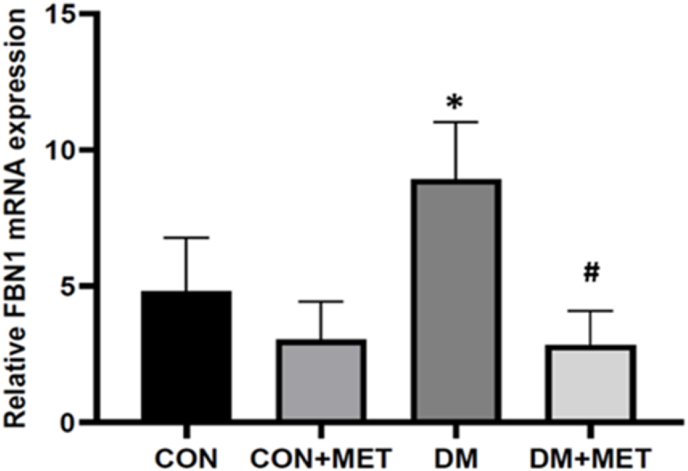
Effect of metformin consumption on FBN1 mRNA expression in different groups Data are expressed as mean ± SEM. CON group: control rats group; DM group: HFD/STZ-induced diabetic rats group; DM + MET rats: HFD/STZ-induced diabetic rats supplemented with metformin treatment; CON + MET: control rats group supplemented with metformin treatment ∗ P = 0.0053, DM vs. CON # P = 0.0001, DM + MET vs. DM.

In the last step, we put the synthesized cDNA into the thermal cycler machine (Applied Biosystems) using the master mix SYBR Green solution (ParsToos, Iran). The primers used in the qRT-PCR reaction are shown in Table 1. The LinReg PCR software was used to calculate the PCR efficiency. Fig. 1 shows the relative FBN1 gene expression (Ruijter et al., 2009).

**Table 1:** Primers used for the analysis of gene expression in adipose tissue.

	Primer sequence	
FBN1	F	5′-GCTCCTACAGATGCGAATGC-3′
	R	5′-CAACGGCTTCACACACTGG-3′
β-Actin	F	5′-ACATCCGTAAAGACCTCTATGCCAACA-3′
	R	5′-GTGCTAGGAGCCAGGGCAGTAATCT-3′

**FBN1:** Fibrillin-1; **β-Actin:** beta-actin; **F**: forward; and **R**: reverse.

### 2.12 Statistical analysis

Statistical analyses were performed using SPSS 20 software. Data distribution was first checked using the Kolmogorov-Smirnov test. If the data were normally distributed, the ANOVA statistical test was used to compare the variables. If the data were not normally distributed, equivalent non-parametric tests were used. The Kruskal-Wallis test was used to compare non-parametric quantitative data, and the Mann-Whitney test was used to compare non-parametric qualitative data. A significance level of less than 0.05 was considered statistically significant.

## 3 Results

### 3.1 Rat's weights

As shown in Table 2, at the end of the study, the DM group had significantly lower weights compared to the CON group (P < 0.0001). The CON + MET group also experienced significant weight loss compared to the CON group (P < 0.0001), likely due to the use of metformin. The DM + MET group exhibited substantial weight loss compared to both the CON and CON + MET groups (P < 0.05), suggesting a combined effect of diabetes and metformin treatment.

**Table 2:** Comparison (mean ± SEM) of the FBS, the lipid profiles, the Body weight, and the HOMA-IR in different groups of Wistar rats.

Parameters	CON	CON + MET	DM	DM + MET	Significance (P-values)
Body weight (gr)	286.9 ± 12.02	2.806^a^ 252.1 ±	230 ± 4.065^b^	204.5 ± 5.590^d^	• aP < 0.0001 • bP < 0.0001 • dP < 0.01
FBG (mg/dl)	63.25 ± 2.358	53.25 ± 2.717	273.5 ± 16.05^b^	99.13 ± 11.32^c^	• bP < 0.0001 • cP < 0.0001
FIN (m IU/L)	10.01 ± 0.71	8.134 ± 0.27	7.826 ± 0.26^b^	8.335 ± 0.55	• bP = 0.0254
TG (mg/dl)	98.5 ± 17.40	111.3 ± 7.2	136.0 ± 11.54	70.57 ± 5.075^c^	• cP = 0.0015
TC (mg/dl)	73.67 ± 7.17	57.17 ± 4.48	89.71 ± 5.02	74.71 ± 4.31	
HDL (mg/dl)	34 ± 0.93	29.67 ± 1.820	48.86 ± 2.65^b^	46.43 ±2.959	• bP = 0.0012
LDL (mg/dl)	21.00 ±3.276	14.33 ± 1.145	15.29 ± 1.65	15 ± 0.78	
HOMA-IR	1.5 ± 0.073	0.99 ± 0.079	5.204 ± 0.494^b^	2.141 ± 0.361^c^	• bP < 0.0001 • cP < 0.0001

The body weight (BW), fasting blood glucose (FBG), fasting insulin (FIN), total cholesterol (TC), triglycerides (TG), high-density lipoprotein-cholesterol (HDL), low-density lipoprotein (LDL). Data are expressed as mean ± SEM. CON group: control rats group; DM group: HFD/STZ-induced diabetic rats group; DM + MET rats: HFD/STZ-induced diabetic rats supplemented with metformin treatment.

a P < 0.0001 in comparison with the CON group.

b P < 0.05 in comparison with the CON group.

c P < 0.002 in comparison with the DM group.

d P < 0.01 in comparison with the CON + MET and the CON group.

### 3.2 Biochemical findings

#### 3.2.1 Blood glucose levels

As displayed in Table 2, fasting blood sugar levels decreased in the metformin-consuming groups (CON + MET, DM + MET) as expected. However, this decrease was not significant in the CON + MET group compared to the CON group (P > 0.05). In contrast, the DM + MET group exhibited a significant decrease in fasting blood sugar compared to the DM group (P < 0.0001). Additionally, the DM group had significantly higher fasting blood sugar levels compared to the CON group (P < 0.0001).

#### 3.2.2 Lipid profile

HDL levels were significantly higher in the DM group than in the CON group (P = 0.0012). Compared to the CON group, the CON + MET group had lower total cholesterol (TC), HDL, and LDL but higher triglycerides (TG). However, these differences were not statistically significant (p > 0.05). In the DM + MET group, TC, TG, HDL, and LDL were reduced compared to the DM group. The only significant reduction was in TG (P = 0.0015) (Table 2).

#### 3.2.3 HOMA-IR

Insulin resistance was significantly higher in the DM group compared to the control group (P < 0.0001). In the DM + MET group, insulin resistance was significantly lower compared to the DM group (P < 0.0001) (Table 2).

#### 3.2.4 FBN1 gene expression

FBN1 gene expression was significantly higher in the DM group than in the CON group (P = 0.0053). Additionally, metformin treatment decreased the expression of FBN1 in the CON + MET group compared to the CON group (P = 0.0053), and it also decreased the expression in the DM + MET group compared to the DM group (P = 0.0001) (Fig. 1).

#### 3.2.5 Asprosin levels

**Fig. 2:**
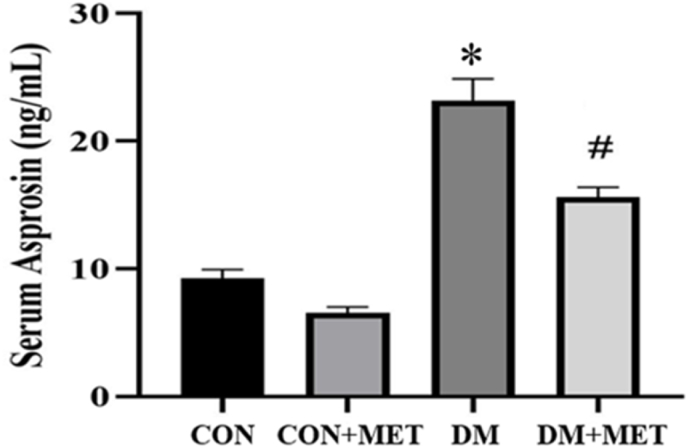
Effect of metformin consumption on different groups Data are expressed as mean ± SEM. CON group: control rats group; DM group: HFD/STZ-induced diabetic rats group; DM + MET rats: HFD/STZ-induced diabetic rats supplemented with metformin treatment; CON + MET: control rats group supplemented with metformin treatment ∗P < 0.0001, DM vs. CON and CON + MET ∗P < 0.01, DM + MET vs. CON and CON + MET. #P = 0.0003, DM + MET vs. DM.

Asprosin levels increased in the DM group, with this increase being significant compared to both the CON and CON + MET groups (P < 0.0001). Metformin significantly decreased serum asprosin levels in the CON + MET group compared to the CON group (P < 0.0001) and in the DM + MET group compared to the DM group (P = 0.0003) (Fig. 2).

## 4 Discussion

Our aim in this study was to investigate the effect of four weeks of metformin consumption on FBN1 gene expression in white adipose tissue and serum asprosin levels. In this study, we found that metformin, in addition to improving chemical biomarkers in the treatment groups, decreased the expression of FBN1 in white adipose tissue. This, in turn, led to a decrease in serum levels of asprosin in the DM + MET group.

Asprosin is a hormone primarily secreted from white adipose tissue during fasting conditions. The serum level of asprosin is notably higher in individuals with diabetes (Romere et al., 2016; Gozel and Kilinc, 2021). The concentration of asprosin, like leptin, increases with fasting and decreases with refeeding (Duerrschmid et al., 2017). Increased circulating asprosin in patients with T2DM may be a risk factor associated with the development of T2DM and may be involved in the pathogenesis of lipid disorders (Zhang et al., 2019). Metformin, a drug with a good safety profile, is commonly used as the initial treatment for T2DM (McCreight et al., 2016). Metformin lowers blood glucose through different mechanisms, with its greatest effect being the inhibition of gluconeogenesis and reduction of hepatic glucose output (Shaw et al., 2005; Fullerton et al., 2013). Conversely, asprosin increases hepatic glucose production via the cAMP/PKA pathway, thereby increasing hepatic glucose output and having a protective effect against hypoglycemia (Romere et al., 2016; Greenhill, 2016).

This study found that the DM group had significantly higher serum asprosin levels compared to both the CON and CON + MET groups (P < 0.0001). Additionally, the DM + MET group had significantly lower serum asprosin levels compared to the DM group (P = 0.0003).

In 2021, Al-Daghr et al. conducted a human study that found higher serum asprosin levels in obese and diabetic subjects compared to control subjects (Al-Daghri et al., 2022), supporting o ur own findings. Another study from 2019 by Kattawy et al. suggested that anti-asprosin could potentially be used as a protective agent against obesity and T2DM (El Kattawy and Ashour, 2019).

Additionally, Zhang et al. discovered that elevated asprosin levels were associated with increased blood glucose, insulin resistance, and waist circumference, indicating that asprosin could be a biomarker for early diabetes diagnosis (Zhang et al., 2019). While some studies propose that asprosin may act as a protective hormone against hypoglycemia, similar to a counterregulatory hormone, the majority of studies suggest that asprosin actually contributes to the development of diabetes (Romere et al., 2016; Greenhill, 2016). Metformin has a diverse mechanism of action, and this study aimed to determine its effect on FBN1 gene expression. Previous studies have shown that metformin can decrease serum asprosin levels in diabetic individuals (Gozel and Kilinc, 2021). However, no study has investigated the effect of metformin on FBN1 gene expression in adipose tissue, the primary organ that secretes asprosin, and compared serum asprosin levels in diabetic and non-diabetic groups.

Our findings indicate that metformin can reduce FBN1 gene expression in both the CON + MET group compared to the CON group and the DM + MET group compared to the DM group. However, the effect was statistically significant only in the DM + MET group compared to the DM group (P = 0.0001).

In contrast to a 2021 study conducted on mice (Miao et al., 2021), our study found that FBN1 gene expression was significantly increased in the diabetic mice group compared to the control group (P = 0.0053).

Metformin exerts various effects on other biochemical parameters, and these effects may be partly mediated by changes in asprosin levels. Asprosin can cross the blood-brain barrier and bind to agouti-related peptide (AgRP+)-expressing neurons (neurons that produce agouti-related protein to increase food intake and decrease energy expenditure) via a G-protein (Gαs)-cAMP-dependent pathway. This interaction may contribute to weight loss in metformin-consuming groups, as metformin reduces appetite by decreasing asprosin levels (Shabir et al., 2021).

Rats in both the CON and DM groups experienced weight loss after metformin administration. This reduction was statistically significant in the CON + MET group compared to the CON group (P < 0.0001) and not significant in the DM + MET group compared to the DM group.

Research has demonstrated that asprosin binds to the OLFR734 receptor on the liver cell membrane, enhancing glucose production via a cAMP-PKA pathway (Romere et al., 2016; Li et al., 2019). Consequently, the reduction in asprosin levels observed in our study may contribute to the glucose-lowering effects of metformin.

We observed that metformin administration decreased asprosin levels and improved insulin resistance in both the diabetic and control groups. In a study conducted on humans, it was observed that asprosin levels in patients with T2DM are higher than in healthy individuals (Naiemian et al., 2020). The result of this experiment was consistent with our findings.

Metformin use exhibited contrasting effects on triglycerides. Metformin was associated with an increase in triglycerides in the CON + MET group compared to the CON group, but the DM + MET group compared to the DM group showed the opposite effect. Furthermore, a human study demonstrated that newly diagnosed T2DM patients had higher triglyceride levels at the time of diagnosis compared to controls. Notably, these triglyceride levels decreased with metformin treatment (Gozel and Kilinc, 2021).

### 4.1 Limitation of study

Our study has some limitations including:

Animal Model: While our study used a well-established rodent model, the findings might not fully translate to human physiology due to species differences. Human studies are necessary to confirm the therapeutic potential of metformin on asprosin levels and FBN1 gene expression.

Duration of Treatment: The treatment duration was limited to four weeks. Long-term studies are essential to understand the chronic effects of metformin on asprosin and FBN1 gene expression.

Single Dose of Metformin: We used a single dosage regimen of metformin (400 mg/kg/day). Future research should explore varying dosages to determine the optimal therapeutic dose and its effects on asprosin and FBN1 gene expression.

Lack of Functional Analysis: Our study focused on biochemical and molecular endpoints. Functional analysis of insulin signaling and glucose tolerance was not performed, which could provide a more comprehensive understanding of metformin's effects.

## 5 Conclusion

This study demonstrated that metformin administration in the diabetic rats significantly reduced both FBN1 gene expression and serum asprosin levels compared to the controls. These findings suggest that metformin's ability to lower asprosin and FBN1 expression may contribute to its effectiveness in managing diabetes. Further studies are warranted to confirm this effect.

## CRediT authorship contribution statement

**Ali Dashtkar:** Conceptualization, Methodology, Study design, Validation, Formal analysis, Investigation, Data curation, Writing – original draft, Visualization. **Mansour Karajibani:** Methodology, Study design, Formal analysis, Resources, Writing – review & editing, Supervision, Funding acquisition. **Mohsen Saravani:** Methodology, Software, Validation, Investigation, Visualization, Supervision. **Roya zanganeh:** Investigation, Data curation. **Hamed Fanaei:** Conceptualization, Methodology, Study design, Software, Validation, Formal analysis, Investigation, Data curation, Writing – original draft, Writing – review & editing, Supervision, Visualization.

## Ethics approval statement

This study was approved by Ethics Committee of Zahedan University of Medical Sciences (ethical code: IR.ZAUMS.AEC.1401.014).

## Data availability

The data used to support the findings of this study are available from the corresponding author upon request.

## Funding Sources

Financial support for the study was conducted by the Office of Vice-Chancellor for Research and Information Technology of 10.13039/501100004847Zahedan University of Medical Sciences (Grant No. 10835).

## Declaration of Competing interest

All authors declare that they have no actual or potential conflict of interest.

## Data Availability

Data will be made available on request.
